# Ionic Liquid-Modified Porous Organometallic Polymers as Efficient and Selective Photocatalysts for Visible-Light-Driven CO_2_ Reduction

**DOI:** 10.34133/2020/9398285

**Published:** 2020-09-25

**Authors:** Zhi-Hua Zhou, Kai-Hong Chen, Song Gao, Zhi-Wen Yang, Liang-Nian He

**Affiliations:** State Key Laboratory and Institute of Elemento-Organic Chemistry, College of Chemistry, Nankai University, Tianjin 300071, China

## Abstract

In the photoreduction of CO_2_ to CO, the competitive H_2_ evolution is always inevitable due to the approximate reduction potentials of H^+^/H_2_ and CO_2_/CO, which results in poor selectivity for CO production. Herein, imidazolium-type ionic liquid- (IL-) modified rhenium bipyridine-based porous organometallic polymers (Re-POMP-IL) were designed as efficient and selective photocatalysts for visible-light CO_2_ photoreduction to CO based on the affinity of IL with CO_2_. Photoreduction studies demonstrated that CO_2_ photoreduction promoted by Re-POMP-IL functioning as the catalyst exhibits excellent CO selectivity up to 95.5% and generate 40.1 mmol CO/g of Re-POMP-IL1.0 (obtained by providing equivalent [(5,5′-divinyl-2,2′-bipyridine)Re(CO)_3_Cl] and 3-ethyl-1-vinyl-1*H*-imidazol-3-ium bromide) at 12 h, outperforming that attained with the corresponding Re-POMP analogue without IL, which highlights the crucial role of IL. Notably, CO_2_ adsorption, light harvesting, and transfer of photogenerated charges as key steps for CO_2_RR were studied by employing POMPs modified with different amounts of IL as photocatalysts, among which the CO_2_ affinity as an important factor for POMPs catalyzed CO_2_ reduction is revealed. Overall, this work provides a practical pathway to improve the CO_2_ photoreduction efficiency and CO selectivity by employing IL as a regulator.

## 1. Introduction

Visible-light-driven reduction of CO_2_ to fuel and/or chemicals provides a promising solution to solve both the environmental problems and the increased energy requirements [1–5]. As a typical product of CO_2_ reduction reaction (CO_2_RR), carbon monoxide (CO) is especially valuable in chemical industry because of its usage as the feedstock for a variety of carbon-based fuels [6, 7]. For photoreduction of CO_2_ to CO, a proton source is always involved, producing the competitive H_2_ evolution accompanied with the CO generation. In fact, the H_2_ formation derived from protons trapping electrons is more favorable than the CO production from CO_2_RR by comparing their reduction potentials [3]. Therefore, the development of photocatalysts capable of disfavouring the H_2_ evolution in an attempt to enhance the efficiency and CO selectivity in CO_2_RR is urgently needed.

For CO_2_ photoreduction to CO, rhenium complexes of *fac*-[Re^I^(N^N)(CO)_3_X] (N^N=diimine, X=Cl, Br) as photocatalysts have gained long-term attention because of their excellent performance [8–16]. However, the notorious instability of *fac*-[Re^I^(N^N)(CO)_3_X] deriving form bimetallic decomposition greatly limits their application. Porous organic polymers (POPs), a type of polymer porous materials with micropores and/or mesopores formed by covalent bonds of organic structural units, feature with advantages of good chemical stability and easily tailored structures [17–20]. To date, several rhenium-metalated conjugated microporous polymers have been designed and demonstrated to be stable photocatalysts for CO_2_ photoreduction [21–25]. Even though, the reported Re(I)-metalated porous materials, commonly fabricated by post modification, namely, polypyridine linkers are firstly constructed and then coordinate with Re(CO)_5_Cl [21–25]. Unfortunately, with post modification, incomplete coordination and random metal anchoring are difficult to avoid [26], resulting in compromised catalytic performance and/or inevitable H_2_ evolution in most cases. Comparatively, direct polymerization of metal complexes to prepare porous organometallic polymers (POMPs) can maximally maintain the structures of molecular catalysts, meanwhile, ensure uniform dispersion of metal sites in the porous matrix [26]. On the other hand, as the photocatalysts for CO_2_RR, characteristics with good CO_2_ adsorption, remarkable light-harvesting property, and efficient separation of photogenerated electrons and holes will be desirable [27]. However, it will be a time-consuming work to design an efficient photocatalyst for CO_2_RR, because there is no panacea to meet all of these features. Hence, locating the key factors to CO_2_RR will be helpful to solve this problem.

Ionic liquids (ILs), composed of cations and anions, have been widely applied in CO_2_ capture and conversion [28–33]. For facilitated CO_2_ absorption, IL has been directly added into the photocatalytic CO_2_ systems, which are proven to accelerate the process of CO_2_ to CO conversion and inhibits H_2_ generation [34, 35]. In addition, the ionic group on the second coordination sphere, i.e., ionic second coordination sphere, has been proven to be promoting CO_2_ photoreduction [16]. Therefore, we speculate ILs as regulators that can be covalently introduced into porous materials to enhance their photocatalytic performance.

**Figure 1:**
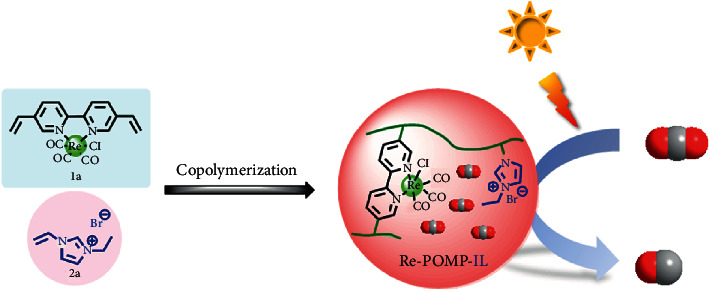
The IL-modified Re-based POMP for CO_2_ photoreduction to CO.

Herein, the novel IL-modified Re-based POMP (Re-POMP-IL) through the simple and scalable copolymerization of Re bipyridine complex and IL was designed and synthesized as shown in Figure 1. By revealing the performance by employing Re-POMP-IL as the photocatalyst for CO_2_ reduction to CO, the promoting effect of IL on photocatalytic activity and CO selectivity is highlighted. In addition, the influence of CO_2_ adsorption ability, optical property, and lifetime for photogenerated charges on the performance of these polymers in photocatalytic CO_2_RR was systematically evaluated by tuning the amount of IL in the preparation of Re-POMP-IL, in which the important factor of efficiency enhancement ascribed to the easy CO_2_ adsorption of Re-POMP-IL was exposed.

## 2. Results

**Figure 2:**
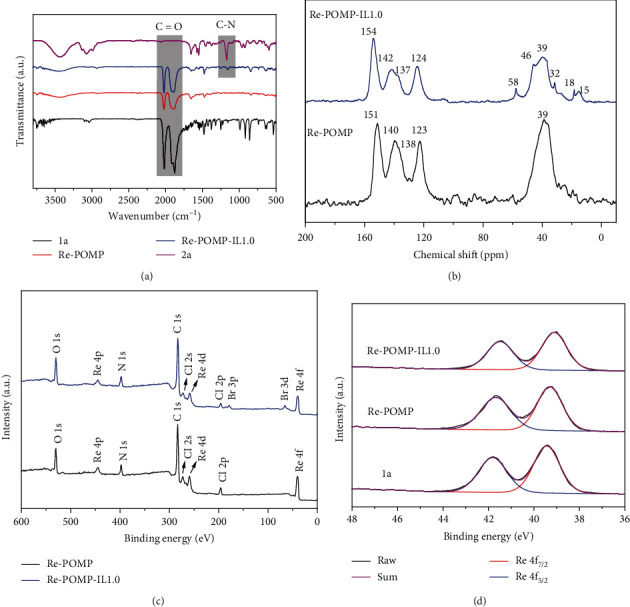
(a) FT-IR spectra of Re-POMP, Re-POMP-IL1.0, and their precursors. (b) Solid-state ^13^C CP/MAS NMR spectra of Re-POMP and Re-POMP-IL1.0. (c) XPS survey spectra of Re-POMP and Re-POMP-IL1.0. (d) XPS spectra of Re 4f in 1a, Re-POMP, and Re-POMP-IL1.0.

As shown in Figure 1, Re-based polymers used in this work can be easily prepared through the solvothermal copolymerization of [(5,5′-divinyl-2,2′-bipyridine)Re(CO)_3_Cl] (**1a**) and 3-ethyl-1-vinyl-1*H*-imidazol-3-ium bromide (**2a**). For Re-POMP-IL1.0, an equivalent **1a** and **2a** were used. For comparison, Re-POMP without IL moiety was also synthesized by self-polymerization of **1a**. The synthesized polymers, attained as yellow solids, are stable in air and insoluble in water and organic solvents (e.g., CH_3_CN). The chemical structures of Re-POMP and Re-POMP-IL1.0 were characterized by FT-IR and solid-state ^13^C CP/MAS NMR technique. As exhibited in Figure 2(a), signals between 2025 and 1870 cm^−1^ are ascribed to the stretching vibration of CO ligand coordinated with Re(I). Compared with Re-POMP, the occurrence of the new peak at 1157 cm^−1^ in the FT-IR spectrum of Re-POMP-IL1.0 arises from the C-N bond vibration of covalently linked **2a**. In the solid-state ^13^C CP/MAS NMR spectra (Figure 2(b)), chemical shifts ranging from 154 to 123 ppm indicate the signals of bipyridyl moieties, and the disappearance of signals of vinyl groups accompanying with the appearance of a new peak at 39 ppm demonstrates the successful polymerization of monomers [36]. The other peaks at 58, 46, 32, 18, and 15 ppm are assigned to the carbons of alkyl chains of polymerized **2a**. Similar morphologies of Re-POMP and Re-POMP-IL1.0 were observed by scanning electron microscopy (SEM) (Figure S4, see Supplementary Materials) and transmission electron microscopy (TEM) (Figure S4, see Supplementary Materials) measurements, which indicates the addition of IL unit does not affect the morphology of Re-POMP. The thermal stability of Re-POMP and Re-POMP-IL1.0 was measured through thermogravimetric analysis (TGA), and no obvious weight loss was found until temperature was above 300°C (Figure S5, see Supplementary Materials). Additionally, power X-ray diffraction (PXRD) pattern of Re-POMP-IL1.0 exhibits the broad diffraction peak (Figure S6, see Supplementary Materials), revealing the amorphous character of the polymer.

Subsequently, the elemental compositions of Re-POMP and Re-POMP-IL1.0 were identified by X-ray photoelectron spectroscopy (XPS). As shown in Figure 2(c), elements including C, N, O, Cl, and Re exist in the survey spectrum of Re-POMP. For Re-POMP-IL1.0, element of Br is also detected because of the introduction of **2a**. The high-resolution Re 4f spectra display two peaks belonging to Re 4f_5/2_ and Re 4f_7/2_, respectively (Figure 2(d)). Similar chemical environment of Re in **1a**, Re-POMP and Re-POMP-IL1.0 was observed, indicating that the polymerization does not destroy the coordination of Re(I) complex. Compared with Re-POMP, both of Re 4f peaks shift to lower binding energies for Re-POMP-IL1.0, indicating the increased Re electron density after the installation of IL. The high electron density on Re atom may facilitate the interaction between Re and CO_2_ (vide infra). The accurate metal loading in polymers was further measured by inductively coupled plasma-optical emission spectroscopy (ICP-OES), and the Re contents of 31.0 and 24.4 wt% in Re-POMP and Re-POMP-IL1.0 were detected, respectively, which are basically consistent with the theoretical results. It is noteworthy that this is the highest Re content detected among the reported Re-based porous materials used in CO_2_RR [21–25] (Table fS1, see Supplementary Materials), and such high concentrations of bpy-Re were conceived to dramatically improve their activity in CO_2_ photoreduction.

**Figure 3:**
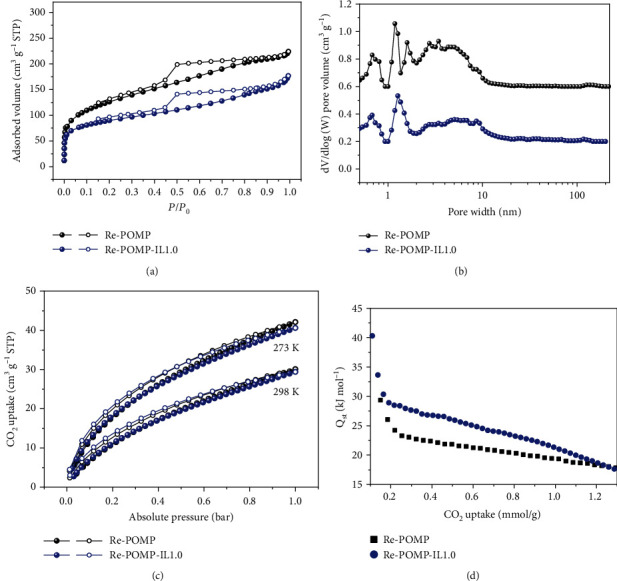
(a) Nitrogen sorption isotherms collected at 77 K. (b) Pore size distributions calculated by NLDFT. (c) CO_2_ uptakes at 273 and 298 K. (d) Isosteric heat of adsorption (*Q*_st_) plots for CO_2_.

The pore structures of polymers were investigated via N_2_ physical sorption at 77 K. As exhibited in Figures 3(a) and 3(b), the N_2_ adsorption/desorption isotherms of Re-POMP and Re-POMP-IL1.0 showed similar characteristics with a combination of type I and type IV patterns [37] with predominant pore sizes ranging from 0.5 to 10 nm, indicating their hierarchical porosity with micro- and mesopores. In fact, hierarchical porous polymers have remarkable advantages over those with microporous structures, profiting from the existence of mesopores facilitates the mass transfer process [17]. Porosity parameters of Re-POMP and Re-POMP-IL1.0 are summarized in Table 1, the Brunauer–Emmett–Teller (BET) surface areas are 452 and 326 m^2^·g^−1^, respectively, and total pore volumes are 0.33 and 0.24 cm^3^·g^−1^, respectively. Clearly, the introduction of **2a** leads to the decrease in BET surface area and total pore volume, possibly due to the blocking of intrinsic pores by flexible chains or Br^−^ of IL [38].

The CO_2_ uptake capacities of Re-POMP and Re-POMP-IL1.0 were also examined (Figure 3(c) and Table 1). A slightly reduced adsorption capacity of Re-POMP-IL1.0 relative to Re-POMP was observed, probably owing to the obvious reduced BET surface area and total pore volume after the introduction of IL. The interaction between polymers and CO_2_ was further evaluated by using the Clausius−Clapeyron equation to calculate the isosteric heat of adsorption (*Q*_st_). As described in Figure 3(d) and Table 1, *Q*_st_ values of Re-POMP and Re-POMP-IL1.0 are 29.3 and 40.3 kJ·mol^−1^, respectively, demonstrating the high CO_2_ affinity of the synthesized polymers. Typically, the larger *Q*_st_ value of Re-POMP-IL1.0 than Re-POMP means the easier CO_2_ capture, likely being attributed to the electrostatic interaction between IL and CO_2_ [39, 40] and the increased Re electron density in Re-POMP-IL1.0.

**Figure 4:**
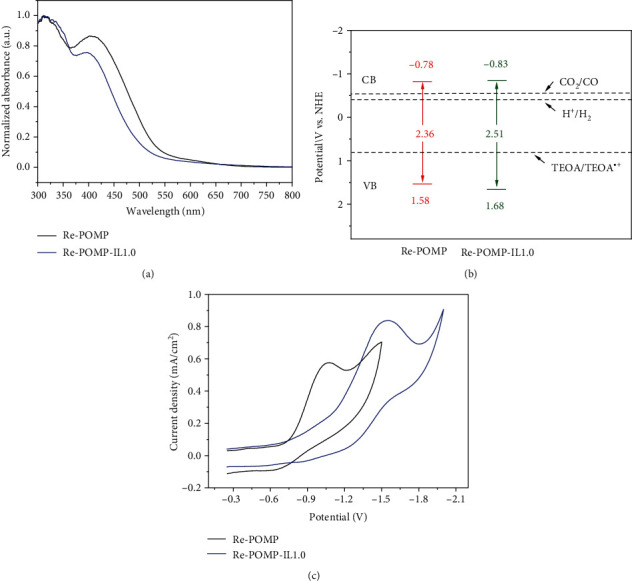
(a) UV/Vis light absorption spectra. (b) CB and VB positions of Re-POMP and Re-POMP-IL1.0. (c) Cyclic voltammograms of Re-POMP and Re-POMP-IL1.0 in acetonitrile with 0.1 M TBAPF_6_ under air at 50 mV s^−1^.

The optical absorption and band gaps of Re-POMP and Re-POMP-IL1.0 were studied by UV/Vis diffuse reflectance spectra as illustrated in Figure 4(a), in which the good visible-light absorption of polymers was observed. Compared with Re-POMP, Re-POMPIL1.0 shows about 50 nm blue shift in absorption onset, probably being ascribed to the existence of IL reduce the conjugated structures [41]. According to their absorption edges, the optical band gaps of Re-POMP and Re-POMP-IL1.0 were obtained from the Tauc plots, in which the band gap is broadened as the presence of IL unit (Figure S7, see Supplementary Materials). In addition, charge separation behavior was investigated through steady-state time-resolved fluorescence spectroscopy (Figure S8, see Supplementary Materials). Fitting results of the fluorescence attenuation curves showed that lifetimes of 80 and 93 ns were calculated for Re-POMP and Re-POMP-IL1.0 (Table 1), respectively, which are sufficient to enable the polymers to exhibit good charge separation. Specifically, the fluorescence lifetime of the polymer is prolonged after IL addition, meaning that Re-POMP-IL1.0 have more free charge to participate in the photoreaction than Re-POMP.

The semiconductor properties and the electronic band positions were estimated by the Mott-Schottky measurements at frequencies of 500 and 1000 Hz (Figure S9, see Supplementary Materials). Re-POMP and Re-POMP-IL1.0 show characteristics of *n*-type semiconductors in view of the positive slopes of Mott-Schottky plots [42]. For *n*-type semiconductors, the bottoms of the conduction band (CB) positions reflecting the LOMO levels are generally close to their flat band potentials [21], which are the intersection values on the abscissa obtained by depicting the plots of C^−2^ values relative to applied potentials. As shown in Figure 4(b), LUMO values (bottoms of conduction band) of -0.78 and -0.83 V vs. NHE for Re-POMP and Re-POMP-IL1.0 were measured, respectively. In combination with the band gaps obtained by Tauc plots, the valence band (VB) of 1.58 and 1.68 V vs. NHE for Re-POMP and Re-POMP-IL1.0 were also calculated, respectively. To further have a knowledge of the redox abilities of polymers, cyclic voltammograms of Re-POMP and Re-POMP-IL1.0 were depicted in Figure 4(c). The reduction potentials of Re-POMP and Re-POMP-IL1.0 were observed at -1.07 and -1.55 V vs. Ag/AgCl (-0.87 and -1.35 V vs. NHE), respectively. Obviously, the more negative reduction potentials of Re-POMP and Re-POMP-IL1.0 than *E*^0^(CO_2_/CO) (-0.53 V vs. NHE, pH=7 in aqueous solution) [43] make the synthesized polymers exhibit great possibility for photoreduction of CO_2_ to CO.

Collectively, the installation of IL into Re-POMP has a little effect on the morphology, while the added IL leads to a decrease in BET surface area so as to affect the CO_2_ adsorption capacity, and simultaneously impede the visible-light harvest. On the other hand, the significantly enhanced CO_2_ affinity and prolonged lifetime of photogenerated charges are found because of the introduction of IL unit. Upon acquiring these features, the performance of IL-modified Re-based porous material as photocatalyst for CO_2_ reduction to CO was evaluated and the decisive factor affecting the CO_2_RR was further elucidated.

**Figure 5:**
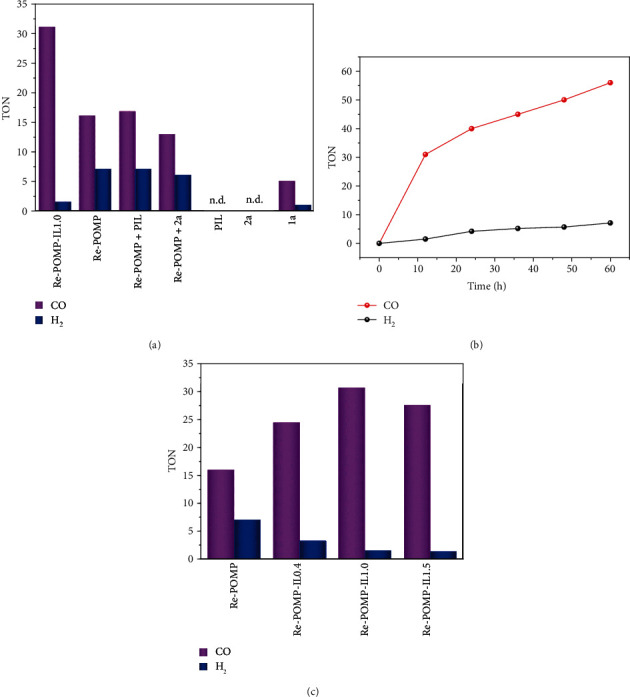
(a) Photocatalytic tests. (b) CO and H_2_ production curves over Re-POMP-IL1.0. (c) TON of CO and H_2_ with POMPs as the photocatalysts.

With Re-POMP-IL1.0 as the photocatalyst, the photocatalytic experiment was firstly carried out under a pure CO_2_ atmosphere (1.0 atm, 298 K) in MeCN solution with triethanolamine (TEOA) as the sacrificial agent and 500 W long-arc Xenon lamp (*λ* ≥ 400 nm) as the light source. As shown in Figure 5(a), CO_2_ can be effectively reduced to CO with a TON value of 30.9 and CO selectivity of 95.4%. In fact, the produced CO amount of 40.1 mmol/g Re-POMP-IL1.0 at 12 h is more than those of reported Re(I)-metalated porous polymers [21–25] (Table S1, see Supplementary Materials), probably benefiting from the relatively high Re content and the hierarchical porosity of Re-POMP-IL1.0. No other reduction products such as HCOOH and MeOH were found, and H_2_ was detected as a by-product owing to the competitive proton reduction [22]. For comparison, employing Re-POMP as the photocatalyst under the consistent conditions only give TON for CO (TON_CO_) of 16.1 and selectivity of 69%. These results can clearly demonstrate that IL as a modifier has the ability to enhance the performance of Re(I)-based porous material. In addition, self-polymerization of **2a** was conducted, in which the polymerized ionic liquid named as PIL was obtained and characterized (Figure S10 and S11, see Supplementary Materials). However, physically mixing of Re-POMP and PIL or IL **2a** cannot achieve the comparable activity to that of Re-POMP-IL1.0 and individual PIL or **2a** as the photocatalyst does not work, meaning that the superior properties of IL-modified porous organometallic polymers are the key to enhance the photocatalytic performance.

In addition, it is noteworthy that these synthesized Re-POMPs exhibited even better activity than the homogeneous one, i.e., **1a** (Figure 5(a)), demonstrating the superiority of pore materials which may provide a unique reaction microenvironment and has the ability to increase the CO_2_ concentration around the catalytic sites [17, 18]. Particularly, POMP exhibits good photostability and Re-POMP-IL1.0 as the photocatalyst for CO_2_ reduction keeps working even if the irradiation time is prolonged to 60 h (Figure 5(b)). At this time, TON_CO_ value is 56 producing CO amount of 73 mmol/g of Re-POMP-IL1.0. Further investigation of the necessity of each component in the catalytic system showed that CO cannot be produced if any of Re-POMP-IL1.0, light source, TEOA, or CO_2_ was absent (Table S2 in Supplementary Materials). The isotopic labeling experiment by employing ^13^CO_2_ for CO_2_RR affords ^13^CO as the major product determined by GC-MS analysis, confirming the CO source originating from CO_2_ (Figure S12, see Supplementary Materials).

For CO_2_RR, light harvesting, transfer of photogenerated charges as well as CO_2_ capture and conversion are key steps [27]. In fact, slightly reduced CO_2_ adsorption capacity, shortened optical absorption range, significantly enhanced CO_2_ affinity, and prolonged lifetime of photogenerated charges of Re-POMP-IL1.0 relative to Re-POMP were observed in this study (see Figures 3(c) and 4(a), Table 1, and Figure S8). The better catalytic performance of Re-POMP-IL1.0 than Re-POMP suggests that the processes of interaction with CO_2_ and separation of photogenerated charges may be closer factors than CO_2_ uptake amount and optical property for POMPs catalyzed CO_2_RR.

In view that the amount of IL in polymer has an effect on the properties, which may affect its photocatalytic activity, Re-POMP-IL0.4 and Re-POMP-IL1.5 were prepared by adjusting the amount of added **2a** in copolymerization using the same synthetic method with Re-POMP-IL1.0. Through ICP-OES, Re contents in Re-POMP-IL0.4 and Re-POMP-IL1.5 were measured as 29.3 and 22.6 wt%, respectively. The N_2_ adsorption/desorption isotherms, pore size distributions, porosity properties, CO_2_ uptake capacities, and excited-state lifetimes of Re-POMP-IL0.4 and Re-POMP-IL1.5 were measured in comparison with those of Re-POMP and Re-POMP-IL1.0 in Figure S13 and Table S3 (see Supplementary Materials). Re-POMP-IL1.5 exhibits much lower surface area than other POMPs, being attributed to the significantly reduced meso- and micropores probably because of the blocking of intrinsic pores by more IL [38]. The higher CO_2_ uptake amounts of Re-POMP-IL0.4 than Re-POMP further highlight the positive effect of IL on CO_2_ absorption, while the reduced CO_2_ uptake amounts of Re-POMP-IL1.0 and Re-POMP-IL1.5 than those of Re-POMP could be probably due to their obviously reduced BET surface area and pore volume. Accompanying with the introduction of IL, the gradually increasing *Q*_st_ values in the order of Re-POMP-IL1.0>Re-POMP-IL0.4>Re-POMP were observed, suggesting the enhanced CO_2_ capture with more IL installed. On the other hand, CO_2_ absorption behavior is also affected by porous properties; thus, a slight reduced *Q*_st_ value of Re-POMP-IL1.5 compared with Re-POMP-IL1.0 may be attributed to its significantly reduced meso- and micropores. The low to moderate *Q*_st_ values of POMPs allow reversible CO_2_ absorption and relatively easy CO_2_ desorption, indicating major physical adsorption rather than chemical adsorption of POMPs for CO_2_ uptake was performed [38, 44]. Moreover, the continually prolonged excited-state lifetimes of POMPs accompanied with the increase of added IL amount were discovered.

Then, Re-POMP-IL0.4 and Re-POMP-IL1.5 were applied to the photocatalytic CO_2_ reduction and both of them exhibits higher activity and CO selectivity than Re-POMP (Figure 5(c)), further illustrating the promoting effect on photocatalytic performance by chemically introducing IL into polymer and simultaneously proving the rationality of the design. In addition, the amount of IL in polymer also has an influence on the activity. Increased TON_CO_ and selectivity of CO were found when advancing the amount of **2a** from 0 to 1.0 equivalent relative to **1a**, probably profiting from the increased IL in polymer can reduce its energy consumption for CO_2_ capture and simultaneously lead to the prolonged excited-state lifetime. However, continuing to increase the amount of **2a** to 1.5 equivalent relative to **1a**, the produced CO began to decrease even though Re-POMP-IL1.5 owns the longest excited-state lifetime among these polymers. On the other hand, the enhanced photocatalytic activity in the order of Re-POMP<Re-POMP-IL0.4<Re-POMP-IL1.5<Re-POMP-IL1.0 was observed (Figure 5(c), Table S3), being consistent with their *Q*_st_ values. These results further elucidate the important effect of CO_2_ affinity on CO_2_RR using POMPs as the photocatalysts. This finding is different from the previous work, in which the high CO_2_ adsorption capacity, broadened optical absorption, and/or facile charge separation are always highlighted [21, 23, 25].

## 3. Discussion

In conclusion, the IL-modified Re-based porous organometallic polymers (Re-POMP-IL) have been designed and developed as effective photocatalysts for CO_2_ reduction to CO. Specifically, the installation of IL (3-ethyl-1-vinyl-1*H*-imidazol-3-ium bromide used in this work) can obviously enhance the photocatalytic activity and CO selectivity. Re-POMP-IL can be obtained through radical copolymerization, which is simple and scalable. Characteristics on pore features, optical properties, redox abilities, and excited-state lifetimes of Re-POMP-IL demonstrated that they have high CO_2_ uptake capacities, visible-light absorption, suitable redox potential, and long enough excited-state lifetimes for CO_2_ reduction. Catalytic activity tests showed that Re-POMP-IL behaves better than Re-POMP in promoting CO_2_ to selectively produce CO, and the competitive H_2_ evolution can be effectively suppressed with CO selectivity up to 95.5%. Investigation on the effect of IL content on the catalytic performance of POMPs shows that Re-POMP-IL1.0 with the largest CO_2_ isosteric heat of adsorption exhibits the best catalytic performance, emphasizing the important role of CO_2_ affinity for POMPs catalyzed CO_2_RR. In a word, these results demonstrate the feasibility of using IL as a regulator to develop efficient and selective photocatalyst for CO_2_ reduction.

## 4. Materials and Methods

### 4.1. Synthesis of [(5,5′-divinyl-2,2′-bipyridine)Re(CO)_3_Cl] (1a).

For the synthesis of **1a**, 5,5′-divinyl-2,2′-bipyridine as ligand was obtained through the cross-coupling of potassium vinyltrifluoroborate with 5,5′-dibromo-2,2′-bipyridine [45] and then coordinated with Re(CO)_5_Cl. Detailed procedure is listed as follows: to a 250 mL three-necked bottle, 5,5′-dibromo-2,2′-bipyridine (0.9420 g, 3 mmol), potassium vinyltrifluoroborate (1.4997 g, 11.2 mmol), Pd(OAc)_2_ (0.0013 g, 0.06 mmol), PPh_3_ (0.0042 g, 0.16 mmol), and Cs_2_CO_3_ (2.7367 g, 8.4 mmol) were sequentially added, and then THF (48 mL) and H_2_O (2 mL) were injected under N_2_ atmosphere. The obtained mixture was refluxed for 16 h. When the reaction finished, the resultant solution was cooled to room temperature and water (50 mL) was added. Then, the mixture was extracted with ethyl acetate (30 mL × 3), and the resulting organic layers were combined. After dried over anhydrous Na_2_SO_4_, the organic extracts were concentrated under vacuum and the residue was purified by chromatography on silica gel with petroleum ether and ethyl acetate (5/1-2/1) as eluents to give 5,5′-divinyl-2,2′-bipyridine.

Then, 5,5′-divinyl-2,2′-bipyridine (0.2083 g, 1 mmol) and Re(CO)_5_Cl (0.3617 g, 1 mmol) were mixed in a 250 mL three-necked bottle. After this, toluene (100 mL) was injected under N_2_ atmosphere. The reaction was carried out under reflux for 16 h. When finished, the resulting mixture was cooled to room temperature, and the yellow precipitate was filtered off, washed with toluene, and dried under vacuum to give **1a**.

5,5'-Divinyl-2,2'-bipyridine [36]: white solid (0.3064 g, 42% yield); m.p.: 79-80°C. IR (neat, KBr): 3091, 3051, 3007, 2992, 1972, 1802, 1626, 1588, 1467, 1361, 1054, 1019, 898, and 850 cm^−1^. ^1^H NMR (400 MHz, CDCl_3_) *δ* 8.67 (s, 2H), 8.38 (d, *J* = 8.1 Hz, 2H), 7.88 (d, *J* = 8.3 Hz, 2H), 6.77 (dd, *J* = 17.6, 11.0 Hz, 2H), 3.90 (d, *J* = 17.6 Hz, 2H), 3.43 (d, *J* = 11.0 Hz, 2H) ppm. ^13^C NMR (101 MHz, CDCl_3_) *δ* 134.99, 147.77, 133.43, 133.28, 132.94, 120.73, and 116.31 ppm. HRMS (ESI): *m*/*z* calcd for C_14_H_13_N_2_H [M + H]^+^: 209.1068; found: 209.1076.

**1a**: yellow solid (0.4295 g, 84% yield). IR (neat, KBr): 3101, 3070, 3040, 2018, 1914, 1877, 1480, 1379, 1251, 918, and 860 cm^−1^. ^1^H NMR (400 MHz, CDCl_3_) *δ* 8.98 (s, 2H), 8.07 (dt, *J* = 8.4, 3.1 Hz, 4H), 6.78 (dd, *J* = 17.6, 11.0 Hz, 2H), 6.03 (d, *J* = 17.6 Hz, 2H), 3.68 (d, *J* = 11.0 Hz, 2H) ppm. HRMS (ESI): *m*/*z* calcd for C_17_H_12_N_2_O_3_Re [M-Cl]^+^: 479.0400; found: 479.0391.

### 4.2. Synthesis of 3-Ethyl-1-Vinyl-1H-Imidazol-3-Ium Bromide (2a) [46]

To a 50 mL Schlenk tube, 1-vinyl-1*H*-imidazole (4.9873 g, 53 mmol) and bromoethane (6.5400 g, 60 mmol) were added. The mixture was refluxed at 70°C for 3 h and then cooled to room temperature to give the white solid. The desired **2a** was obtained after drying the white solid under vacuum at 70°C for overnight.

**2a** [46]: white solid (10.9509 g, 98% yield). IR (neat, KBr): 3133, 3076, 2991, 1655, 1573, 1550, 1170, 961, and 926 cm^−1^. ^1^H NMR (400 MHz, DMSO-*d*_6_) *δ* 9.61 (s, 1H), 8.23 (d, *J* = 1.5 Hz, 1H), 7.98 (s, 1H), 7.32 (dd, *J* = 15.6, 8.8 Hz, 1H), 5.98 (dd, *J* = 15.6, 2.3 Hz, 1H), 5.41 (dd, *J* = 8.7, 2.3 Hz, 1H), 4.24 (q, *J* = 7.3 Hz, 2H), 1.45 (t, *J* = 7.3 Hz, 3H) ppm. ^13^C NMR (101 MHz, D_2_O) *δ* 134.07, 128.26, 122.55, 119.40, 109.20, 45.22, and 14.34 ppm.

### 4.3. Synthesis of Re-POMP

**1a** (200 mg, 0.39 mmol), AIBN (10 mg, 0.06 mmol), and DMF (7 mL) were added to a 25 mL autoclave. Then, the autoclave was sealed and heated at 100°C for 24 h. When finished, the autoclave was cooled to room temperature and the resulting mixture was transferred to a 50 mL conical flask, followed by adding 20 mL ethanol to soak overnight. Then, the obtained mixture was filtered and washed with ethanol until the filtrate was colorless. After the filter cake was dried under vacuum at 70°C for overnight, the yellow solid (185.7 mg) was obtained and named as Re-POMP.

### 4.4. Synthesis of Re-POMP-IL

Take the synthesis of Re-POMP-IL1.0 as an example, **1a** (200 mg, 0.39 mmol), **2a** (78.8 mg, 0.39 mmol), AIBN (10 mg, 0.06 mmol), and DMF (7 mL) were added to a 25 mL autoclave. Then, the autoclave was heated at 100°C for 24 h. When finished, the autoclave was cooled to room temperature and the resulting mixture was transferred to a 50 mL conical flask, followed by adding 20 mL ethanol to soak overnight. Then, the obtained mixture was filtered and washed with ethanol until the filtrate was colorless. After the filter cake was dried under vacuum at 70°C for overnight, the yellow solid (268.3 mg) was obtained and named as Re-POMP-IL1.0.

For the synthesis of Re-POMP-IL0.4 and Re-POMP-IL1.5, their synthetic procedures are similar to that of Re-POMP-IL1.0, except that the amount of **2a** added was adjusted to 0.16 mmol and 0.59 mmol, respectively.

### 4.5. Synthesis of PIL

**2a** (505 mg, 2.5 mmol), AIBN (24.6 mg, 0.15 mmol), and ethanol (5 mL) were added to a 50 mL Schlenk flask under Ar atmosphere. Then, the reaction was conducted at 80°C for 16 h. When finished, the resultant mixture was cooled to room temperature, followed by adding 20 mL ethanol to soak overnight. Then, the obtained mixture was filtered and washed with ethanol (5 × 20 mL). After the filter cake was dried under vacuum at 70°C for overnight, PIL (420.2 mg) was obtained as white solid.

### 4.6. General Procedure for Photocatalytic CO_2_ Reduction

To a 25 mL Schlenk tube, photocatalyst (1 mg), TEOA (1.12 g, 1 mL) and MeCN (3 mL) were successively added. Then, the reaction mixture was sonicated to allow the polymer to disperse evenly in the whole solution. Air in the Schlenk tube was replaced by CO_2_ through the freeze-pump-thaw method. Then, the reaction tube was sealed and placed under a 500 W long-arc Xenon lamp (*λ* ≥ 400 nm) for 12 h at room temperature. The light source was cooled by flowing cooling water before irradiating the reaction tube and the number of moles of photons absorbed by the photocatalyst was measured as 1.4 × 10^−8^ einstein/s (see Supplementary Materials). After the reaction, partial gaseous products (1 mL) were taken from the tube using a syringe and then analyzed by gas chromatography with a TCD detector.

## Figures and Tables

**Table 1:** Porosity properties, CO_2_ uptake capacities, and lifetimes of excited states.

Polymers	*S*_BET_ (m^2^·g^−1^)^a^	*V*_total_ (cm^3^·g^−1^)^b^	CO_2_ uptake (cm^3^·g^−1^)^c^		*Q*_st_ (kJ·Mol^−1^)^d^	*τ*_a_ (ns)^e^
			273 K	298 K		
Re-POMP	452	0.33	42.1	30.1	29.3	80
Re-POMP-IL1.0	326	0.24	40.6	29.4	40.3	93

^a^Specific surface area calculated by using the BET method. ^b^Single point adsorption total pore volume at *P*/*P*_0_ = 0.95. ^c^CO_2_ uptake capacities at 1 bar. ^d^Isosteric heat of adsorption for CO_2_. ^e^The average photoluminescence lifetime of the polymer.

## Supplementary Materials

Supplementary Materials Figure S1: ^1^H and ^13^C NMR of 5,5′-divinyl-2,2′-bipyridine. Figure S2: ^1^H NMR of [(5,5′-divinyl-2,2′-bipyridine)Re(CO)^3^Cl] (**1a**). Figure S3: ^1^H and ^13^C NMR of 3-ethyl-1-vinyl-1*H*-imidazol-3-ium bromide (**2a**). Figure S4: SEM images of (a) Re-POMP and (b) Re-POMP-IL1.0; TEM images of (c) Re-POMP and (d) Re-POMP-IL1.0. Figure S5: TGA curves of Re-POMP and Re-POMP-IL1.0 in nitrogen. Figure S6: powder X-ray diffraction (PXRD) of Re-POMP-IL1.0. Figure S7: Tauc plots of (a) Re-POMP and (b) Re-POMP-IL1.0. Figure S8: fluorescence decay curves of Re-POMP and Re-POMP-IL1.0 in solid/air at room temperature. Figure S9: Mott-Schottky plots of (a) Re-POMP and (b) Re-POMP-IL1.0 in 0.1 M TBABF_6_ acetonitrile solution at 500 and 1000 Hz. Figure S10: FT-IR spectra of PIL and **2a**. Figure S11: SEM image of PIL. Figure S12: GC-MS spectrum of photogenerated ^13^CO under ^13^CO_2_ atmosphere with Re-POMP-IL1.0 as the photocatalyst. Figure S13: (a) nitrogen sorption isotherms collected at 77 K. (b) Pore size distributions calculated by NLDFT. Table S1: comparison of Re content and the produced CO amount of Re-POMP-IL1.0 with the reported Re-based porous polymers and other porous materials. Table S2: control experiments of photoreduction of CO_2_^a^. Table S3: porosity properties, CO_2_ uptake capacities, and lifetimes of excited states of the polymers and photocatalytic results of the polymers.
