# Supplementary material for: Ionic Liquid-Modified Porous Organometallic Polymers as Efficient and Selective Photocatalysts for Visible-Light-Driven CO2 Reduction
Source: Research (Wash D C). 2020 Sep 25;2020:9398285. doi: 10.34133/2020/9398285 (PMC7533041; doi:10.34133/2020/9398285)
Supplement: Supplementary Materials — Figure S1: 1H and 13C NMR of 5,5′-divinyl-2,2′-bipyridine. Figure S2: 1H NMR of [(5,5′-divinyl-2,2′-bipyridine)Re(CO)3Cl] (1a). Figure S3: 1H and 13C NMR of 3-ethyl-1-vinyl-1H-imidazol-3-ium bromide (2a). Figure S4: SEM images of (a) Re-POMP and (b) Re-POMP-IL1.0; TEM images of (c) Re-POMP and (d) Re-POMP-IL1.0. Figure S5: TGA curves of Re-POMP and Re-POMP-IL1.0 in nitrogen. Figure S6: powder X-ray diffraction (PXRD) of Re-POMP-IL1.0. Figure S7: Tauc plots of (a) Re-POMP and (b) Re-POMP-IL1.0. Figure S8: fluorescence decay curves of Re-POMP and Re-POMP-IL1.0 in solid/air at room temperature. Figure S9: Mott-Schottky plots of (a) Re-POMP and (b) Re-POMP-IL1.0 in 0.1 M TBABF6 acetonitrile solution at 500 and 1000 Hz. Figure S10: FT-IR spectra of PIL and 2a. Figure S11: SEM image of PIL. Figure S12: GC-MS spectrum of photogenerated 13CO under 13CO2 atmosphere with Re-POMP-IL1.0 as the photocatalyst. Figure S13: (a) nitrogen sorption isotherms collected at 77 K. (b) Pore size distributions calculated by NLDFT. Table S1: comparison of Re content and the produced CO amount of Re-POMP-IL1.0 with the reported Re-based porous polymers and other porous materials. Table S2: control experiments of photoreduction of CO2a. Table S3: porosity properties, CO2 uptake capacities, and lifetimes of excited states of the polymers and photocatalytic results of the polymers. [file 9398285.f1.doc]

**Supplementary Materials**

**Ionic Liquid-Modified Porous Organometallic Polymers as Efficient and Selective Photocatalysts for Visible-Light-Driven CO_2_ Reduction**

Zhi-Hua Zhou, Kai-Hong Chen, Song Gao, Zhi-Wen Yang, and Liang-Nian He*

*State Key Laboratory and Institute of Elemento-Organic Chemistry, College of Chemistry, Nankai University, Tianjin, 300071 (P. R. China)*

Correspondence should be addressed to Liang-Nian He; [heln@nankai.edu.cn](mailto:heln@nankai.edu.cn)

**Table of contents**

1. **General Information S2**
2. **Supplementary Figures S4**

**Supplementary References S13**

1. **General Information**

**1.1 Materials**

5,5'-dibromo-2,2'-bipyridine and PPh_3_ were purchased from Energy Chemical, Re(CO)_5_Cl (98%), potassium vinyltrifluoroborate (98%), Pd(OAc)_2_ and Cs_2_CO_3_ (99%) were purchased from J&K Scientific Ltd.. CO_2_ (99.99% purity) was purchased from Liquefied Air (Tianjin) Co., Ltd.. THF and toluene as solvent were freshly distilled from sodium benzophenone ketyl under nitrogen.

**1.2 General Instrumentations and Methods**

The Fourier transform infrared spectroscopy (FT-IR) spectra were recorded on a Bruker Tensor27 FT-IR spectrophotometer with KBr pellets. Liquid ^1^H and ^13^C NMR spectra were determined on Bruker 400, wherein CDCl_3_ or DMSO-*d*_6_ or D_2_O were used as solvent. High resolution mass spectrometry was measured using a Varian 7.0 T FTICR-MS by ESI technique. Solid-state ^13^C CP/MAS NMR spectra were recorded on a Varian Infinityplus-400 spectrometer. Thermogravimetric analysis (TGA) were performed on STA449F3 with a heating rate of 10 °C·min^-1^ in range of 25-800 °C under nitrogen atmosphere. The power X-ray diffraction (PXRD) patterns were measured on Rigaku SmartLab operating at (40 kV, 150 mA) with a scan rate of 2^o^/min from 2^o^ to 90^o^. X-ray photoelectron spectroscopy (XPS) was carried out by using Axis Ultra DLD. Re contents in Re-POMP and Re-POMP-IL were determined by inductively coupled plasma optical emission spectrometer (ICP-OES, SpectroBlue), and the samples (4 mg) were digested with a mixture of HNO_3_ (3 mL) and HClO_4_ (0.5 mL) before measurement. Field emission scanning electron microscopy (SEM) images and field emission transmission electron microscopy (TEM) images were obtained on JSM-7500F at an acceleration voltage of 5 kV and Tecnai G2 F20 at an acceleration voltage of 200 kV, respectively. N_2_ and CO_2_ sorption of the polymers were measured by using Micromeritics ASAP 2020. Pore size distributions were obtained from the adsorption branches of the isotherms using the non-local density functional theory (NLDFT) method and isosteric heat of adsorption (Q_st_) for CO_2_ was calculated with the Clausius−Clapeyron equation. Solid UV-Vis spectra were recorded on a dual beam UV-visible spectrophotometer (TU-1901, Beijing). Electrochemical measurements were carried out on a CHI660d electrochemical workstation with an ITO conductive glass coated the synthesized samples (the coated area is 1 cm^2^) as the working electrode, a Ag/AgCl electrode (saturated KCl solution) as the reference electrode, a platinum plate as the counter electrode and 0.1 M TBAPF_6_ in acetonitrile as the electrolyte. The coated samples were prepared by mixing photocatalyst (5 mg) and Nafion solution (30 μL, 5 wt%) in ethanol (1 mL), then dried in air. The measured potentials were calibrated by ferrocene/ferrocenium (Fc/Fc^+^). Fluorescence decay curves and photoluminescence lifetimes were measured on a transient fluorescence spectrometer (FLS1000) with a 450 nm laser excitation.

**1.3 Measurement of the Number of Moles of Photons**

The number of moles of photons was measured according to previous method [S1]. To a 25 mL flask, 2.5 μmol Ru(bpy)_3_Cl_2_, 2.5 μmol 1,9-diphenylanthracene (DPA) and 25 mL acetonitrile were added. The UV-Vis spectra of the Ru(bpy)_3_Cl_2_ and DPA solution were recorded. The initial absorption of Ru(bpy)_3_Cl_2_ and DPA at 372 nm was noted as A_INITIAL_. Then, the sample was irradiated under 500 W Xenon long-arc lamp (λ ≥ 400 nm) for 10 min and recorded a new UV-Vis spectra of sample at 372 nm, which was noted as A_FINAL_. The equation below was used to calculate the moles of consumed DPA.

moles DPA consumed =
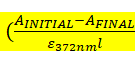
)
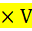


Where A_INITIAL_ and A_FINAL_ are the absorbance of the solution at 372 nm before and after irradiation, respectively;
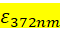
 is the extinction coefficient of DPA at 372 nm in acetonitrile (11000 M^-1^cm^-1^), l is the path length of the cuvette (1 cm), and V is the volume of sample for which the absorption was measured (3 mL). Since the quantum yield (Ф) for Ru(bpy)3Cl2 is known to be 0.019, we used the number of moles DPA consumed to determine the number of moles of photons absorbed by our sample per unit time
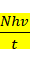
 by applying equation:


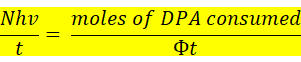


Where Ф is quantum yield of DPA consumption and t is the irradiation in seconds.

1. **Supplementary Figures**

**Fig. S1**. ^1^H and ^13^C NMR of 5,5’-divinyl-2,2’-bipyridine.

**Fig. S2**. ^1^H NMR of [(5,5’-divinyl-2,2’-bipyridine)Re(CO)_3_Cl] (1a).

**Fig. S3**. ^1^H and ^13^C NMR of 3-ethyl-1-vinyl-1*H*-imidazol-3-ium bromide (**2a**).


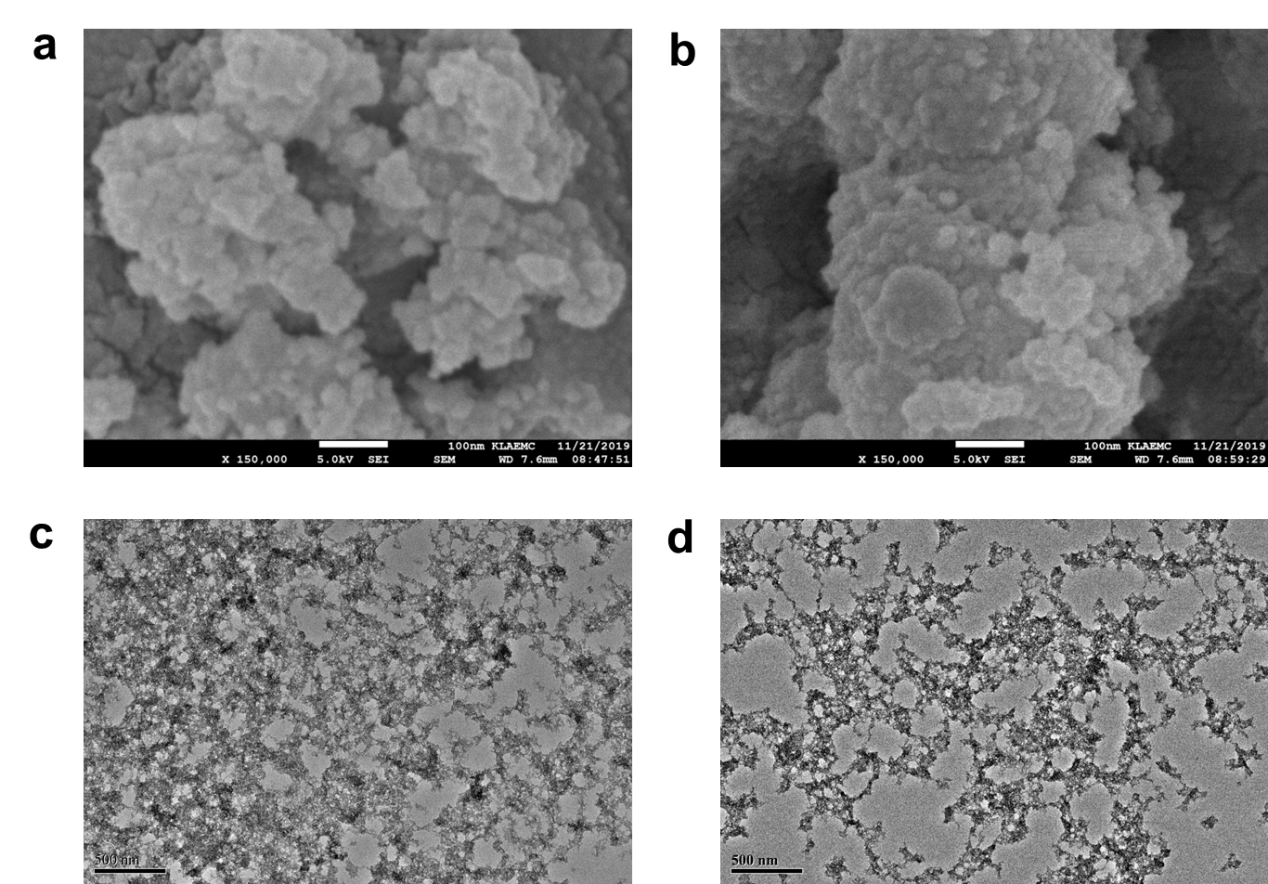


**Fig. S4.** SEM images of (a) Re-POMP and (b) Re-POMP-IL1.0; TEM images of (c) Re-POMP and (d) Re-POMP-IL1.0.


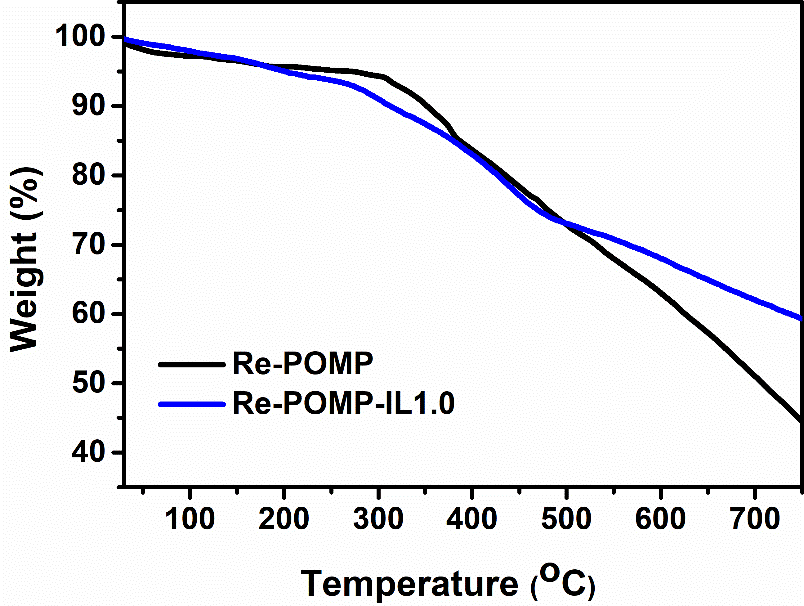


**Fig. S5.** TGA curves of Re-POMP and Re-POMP-IL1.0 in nitrogen.


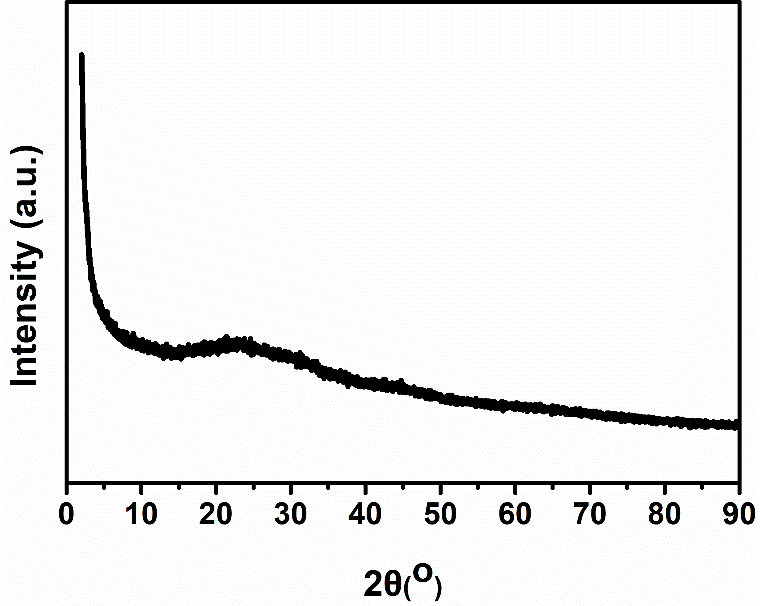


**Fig. S6.** Powder X-ray diffraction (PXRD) of Re-POMP-IL1.0.


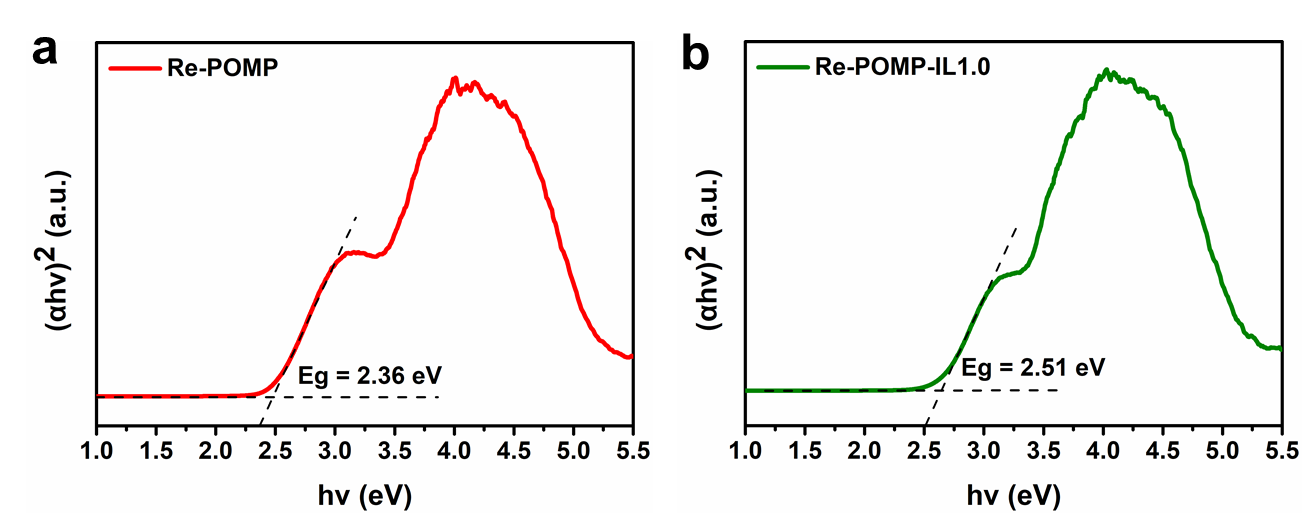


**Fig. S7.** Tauc plots of (a) Re-POMP and (b) Re-POMP-IL1.0.


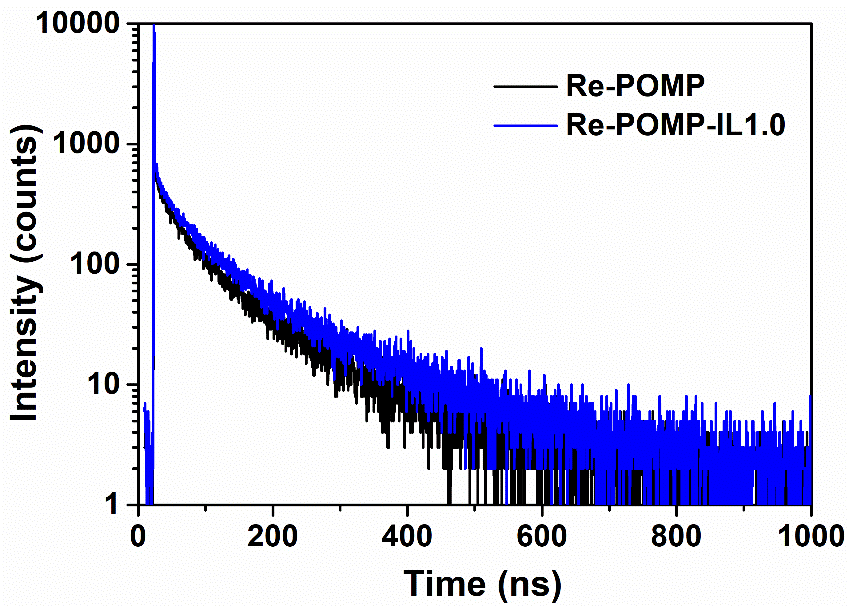


**Fig. S8.** Fluorescence decay curves of Re-POMP and Re-POMP-IL1.0 in solid/ air at room temperature.


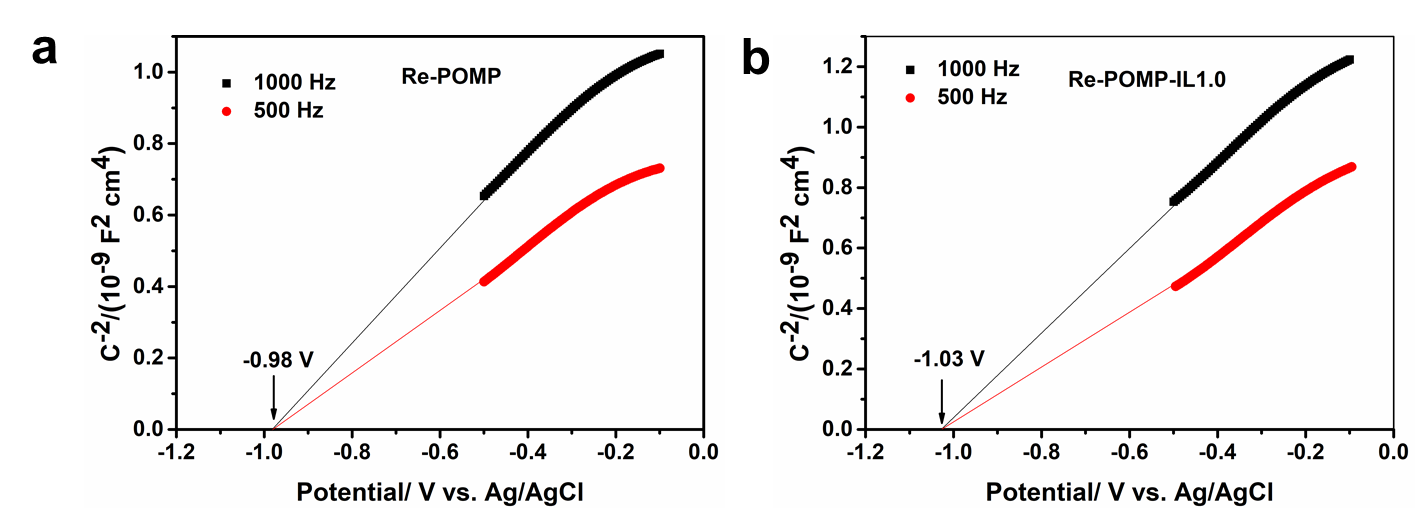


**Fig. S9.** Mott-Schottky plots of (a) Re-POMP, (b) Re-POMP-IL1.0 in 0.1 M TBABF_6_ acetonitrile solution at 500 and 1000 Hz.


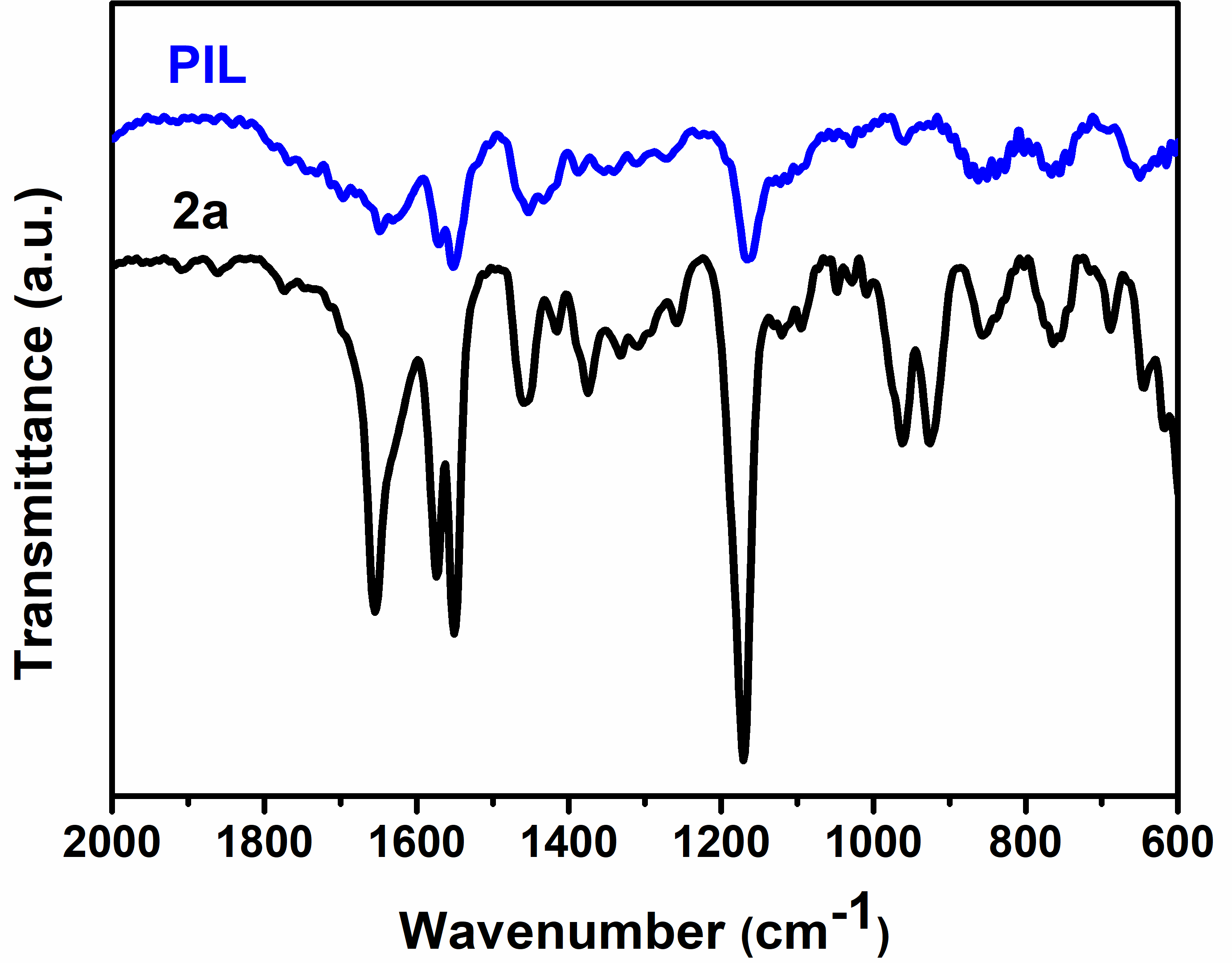


**Fig. S10**. FT-IR spectra of PIL and **2a**.

As shown in Fig. S10, characteristic absorption peaks of alkenyl in **2a** at 1656 (stretching vibration of C=C), 958 and 924 cm^-1^ (wagging vibration of =C-H) disappeared after polymerization indicates the successful preparation of PIL.


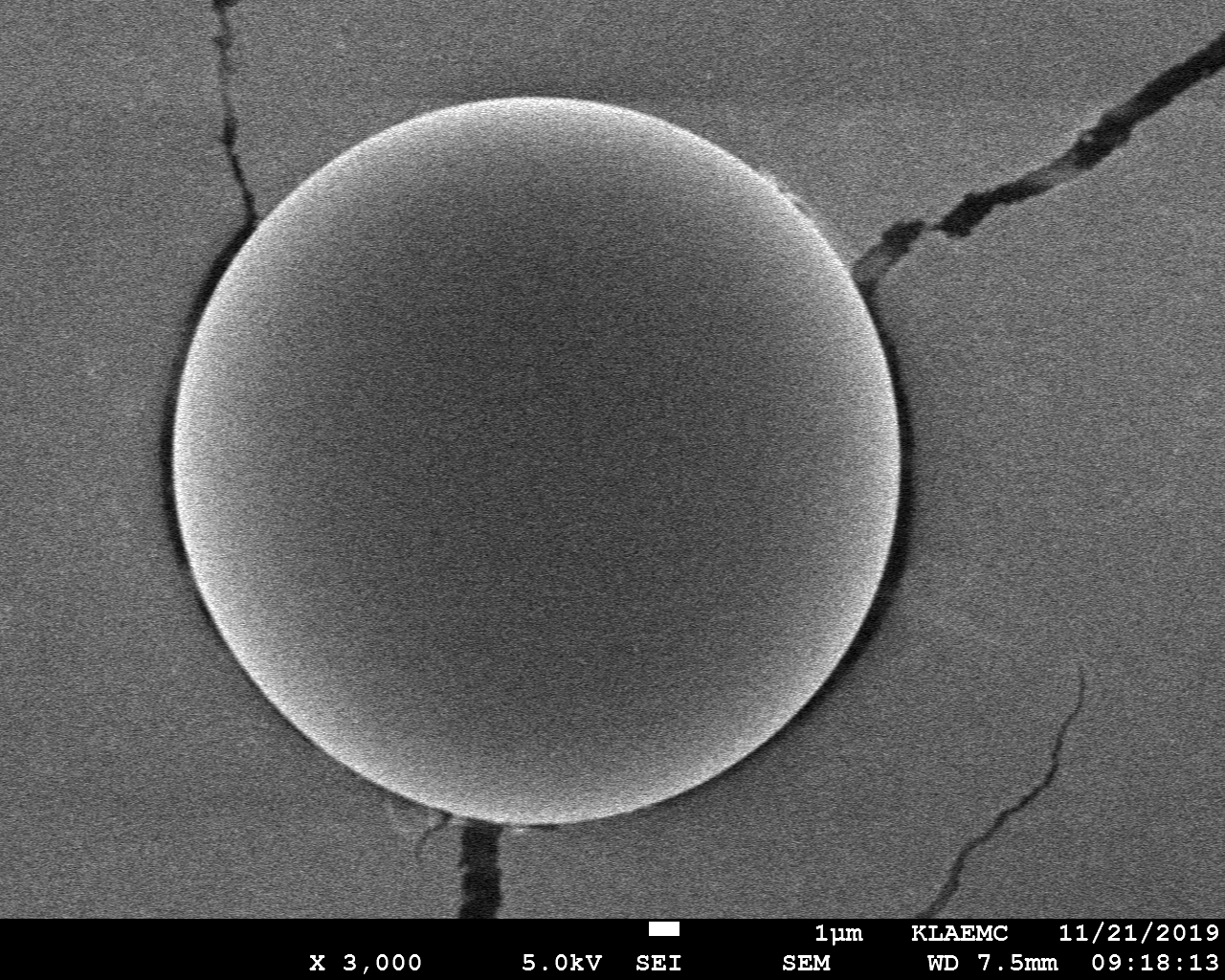


**Fig. S11**. SEM image of PIL.


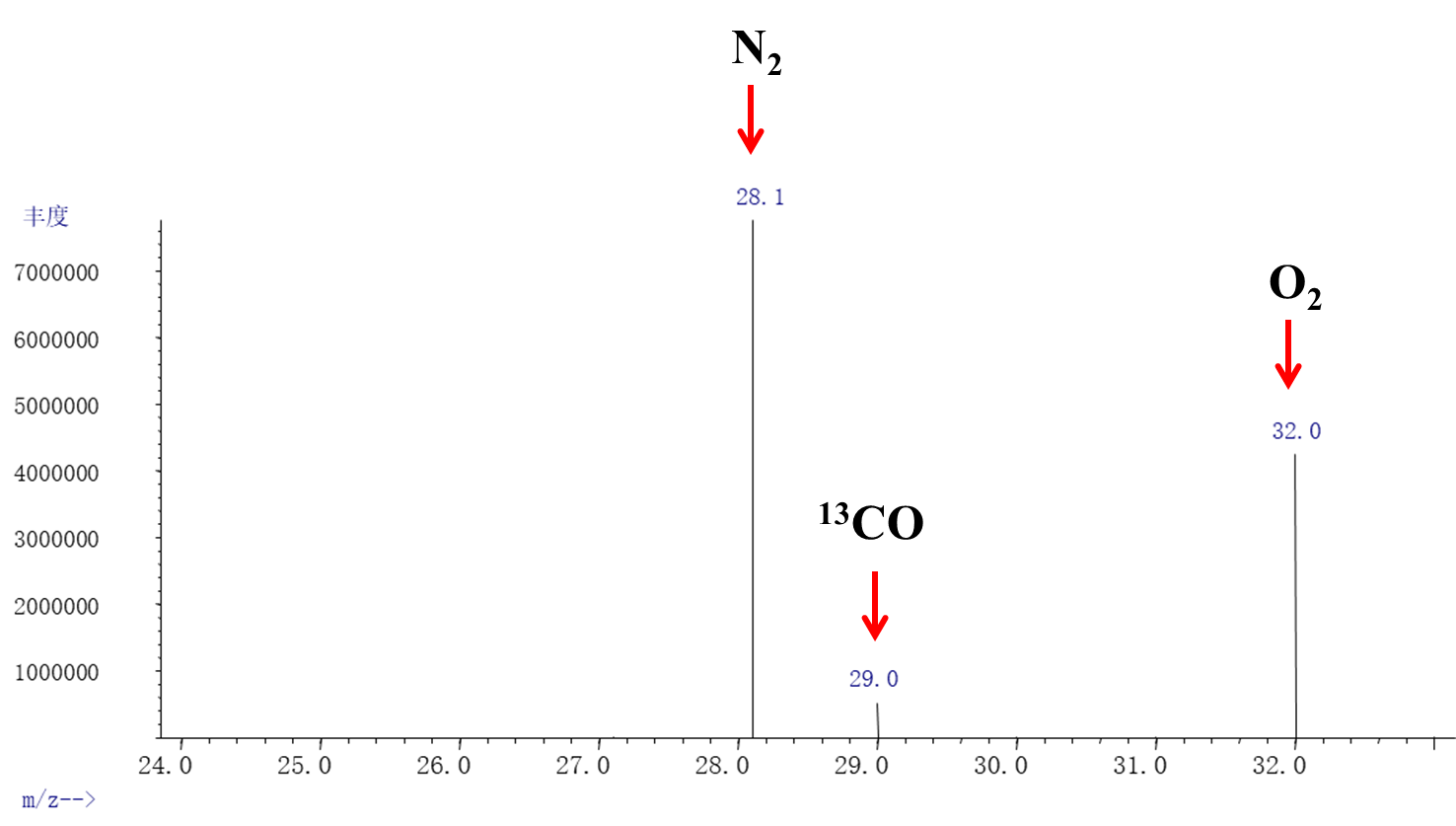


**Fig. S12.** GC-MS spectrum of photogenerated ^13^CO under ^13^CO_2_ atmosphere with Re-POMP-IL1.0 as the photocatalyst.


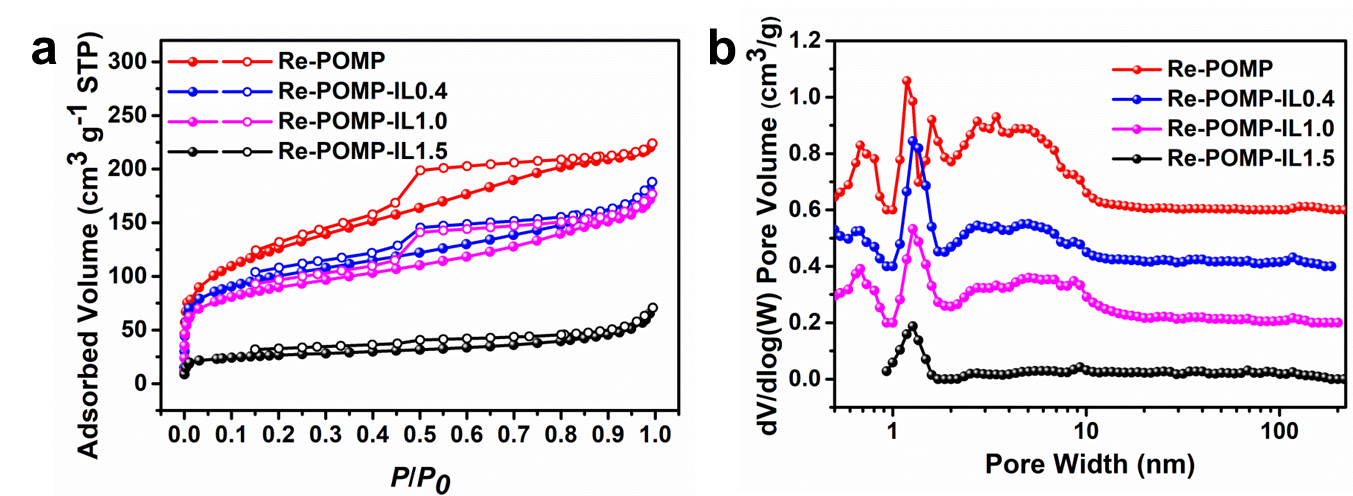


**Fig. S13**. (a) Nitrogen sorption isotherms collected at 77 K. (b) Pore size distributions calculated by NLDFT.

**Table S1.** Comparison of Re content and the produced CO amount of Re-POMP-IL1.0 with the reported Re-based porous polymers and other porous materials.

| Photocatalyst | Re content (wt%) | | Produced CO amount (mmol g^−1^) | Reference |
| --- | --- | --- | --- | --- |
|  | Measured value | Theoretical value |  |  |
| Re-POMP-IL1.0 | 24.4 | 25.9 | 40.1 (12 h) | This work |
| CPOP-30-Re | 16.4 | 23.6 | 6.2 (10 h) | [S2] |
| 1-Re (CMP-BPY-Re) | 14.1 | 22.5 | 3.8 (20 h) | [S3] |
| Re-COF | 5.58 | ~23.4 | ~15 (>20 h) | [S4] |
| Re-Bpy-sp^2^c-COF | 18 | ~17.4 | 18.2 (17.5 h) | [S5] |
| Re-CTF-py | 13.63 | - | 3.5 (10 h) | [S6] |
| Re_0.04_-BPy-PMO | 2.5 | - | 0.75 (24 h) | [S7] |
| Ru_0.04_-Re_0.04_-BPy-PMO | 2.5 | - | 4.03 (24 h) | [S7] |
| Re-BPy_0.3_-NT-Me | 5.4 | - | 5.6 (8 h) | [S8] |
| Eosin Y | - | - | 0.33 (10 h) | [S9] |
| OXD-TPA | - | - | 0.15 (4 h) | [S10] |

**Table S2**. Control experiments of photoreduction of CO_2_*^a^*.

| Entry | Photocatalyst | Light source | Sacrificing reagent | TON(CO)*^b^* | TON(H_2_)*^b^* |
| --- | --- | --- | --- | --- | --- |
| 1 | POMP-IL1.0 | 500 W long-arc Xenon lamp  (λ ≥ 400 nm) | TEOA | 30.9 | 1.5 |
| 2 | - | 500 W long-arc Xenon lamp  (λ ≥ 400 nm) | TEOA | 0 | 0 |
| 3 | POMP-IL1.0 | - | TEOA | 0 | 0 |
| 4 | POMP-IL1.0 | 500 W long-arc Xenon lamp  (λ ≥ 400 nm) | - | 0 | 0 |
| 5*^c^* | POMP-IL1.0 | 500 W long-arc Xenon lamp  (λ ≥ 400 nm) | TEOA | 0 | 0 |
| *^a^* Reaction conditions: photocatalyst (1 mg), sacrificing reagent (1.12 g, 1 mL), MeCN (3 mL), light source, 12 h, CO_2_ (1 bar), 25 mL Schlenk tube. *^b^* Calculated by GC analysis. *^c^* Without CO_2_, under Ar. | | | | | |

**Table S3.** Porosity properties, CO_2_ uptake capacities, lifetimes of excited states and photocatalytic results of the polymers.

| Polymers | S_BET_  (m^2^·g^-1^)^a^ | V_total_  (cm^3^·g^-1^)^b^ | CO_2_ uptake (cm^3^·g^-1^)^c^ | | Q_st_  (kJ/mol)^d^ | τ_a_  (ns)^e^ | Photocatalytic  results^f^ | |
| --- | --- | --- | --- | --- | --- | --- | --- | --- |
|  |  |  | 273 K | 298 K |  |  | TON_CO_ | TON_H2_ |
| Re-POMP | 452 | 0.33 | 42.1 | 30.1 | 29.3 | 80 | 16.1 | 7.2 |
| Re-POMP-IL0.4 | 364 | 0.25 | 74.4 | 52.4 | 37.2 | 82 | 24.6 | 3.2 |
| Re-POMP-IL1.0 | 326 | 0.24 | 40.6 | 29.4 | 40.3 | 93 | 30.9 | 1.5 |
| Re-POMP-IL1.5 | 97 | 0.08 | 32.2 | 23.4 | 39.1 | 114 | 27.7 | 1.3 |

^a^ Specific surface area calculated by using BET method. ^b^ Single point adsorption total pore volume at P/P_0_ = 0.95. ^c^ CO_2_ uptake capacities at 1 bar. ^d^ Isosteric heat of adsorption for CO_2_. ^e^ The average photoluminescence lifetime of the polymer. ^f^ Calculated by GC analysis.

**Supplementary References**

[S1] S. P. Pitre, C. D. McTiernan, W. Vine et al., “Visible-Light Actinometry and Intermittent Illumination as Convenient Tools to Study Ru(bpy)_3_Cl_2_ Mediated Photoredox Transformations,” Scientific Reports, vol. 5, pp. 16397, 2015.

[S2] H.-P. Liang, A. Acharjya, D. A. Anito et al., “Rhenium-Metalated Polypyridine-Based Porous Polycarbazoles for Visible-Light CO_2_ Photoreduction,” ACS Catalysis, vol. 9, no. 5, pp. 3959, 2019.

[S3] W. Liang, T. L. Church, S. Zheng et al., “Site Isolation Leads to Stable Photocatalytic Reduction of CO_2_ over a Rhenium-Based Catalyst,” Chemistry A European Journal, vol. 21, no. 51, pp. 18576, 2015.

[S4] S. Yang, W. Hu, X. Zhang et al., “2D Covalent Organic Frameworks as Intrinsic Photocatalysts for Visible Light-Driven CO_2_ Reduction,” Journal of the American Chemical Society, vol. 140, no. 44, pp. 14614, 2018.

[S5] Z. Fu, X. Wang, A. M. Gardner et al., “A Stable Covalent Organic Framework for Photocatalytic Carbon Dioxide Reduction,” Chemical Science, vol. 11, no. 2, pp. 543, 2020.

[S6] R. Xu, X.-S. Wang, H. Zhao et al., “Rhenium-Modified Porous Covalent Triazine Framework for Highly Efficient Photocatalytic Carbon Dioxide Reduction in A Solid–Gas System,” Catalysis Science & Technology, vol. 8, no. 8, pp. 2224, 2018.

[S7] M. Waki, K.-i. Yamanaka, S. Shirai et al., “Re(bpy)(CO)_3_Cl Immobilized on Bipyridine-Periodic Mesoporous Organosilica for Photocatalytic CO_2_ Reduction,” Chemistry A European Journal, vol. 24, no. 15, pp. 3846, 2018.

[S8] S. Zhang, M. Li, W. Qiu et al., “Heterogeneous Molecular Rhenium Catalyst for CO_2_ Photoreduction with High Activity and Tailored Selectivity in An Aqueous Solution,” Applied Catalysis B: Environmental, vol. 259, pp. 118113, 2019.

[S9] X. Yu, Z. Yang, B. Qiu et al., “Eosin Y-Functionalized Conjugated Organic Polymersfor Visible-Light-Driven CO_2_ Reduction with H_2_O to CO with High Efficiency,” Angewandte Chemie International Edition, vol. 58, no. 2, pp. 632, 2019.

[S10] C. Dai, L. Zhong, X. Gong et al., Triphenylamine Based Conjuated Microporous Polymers for Selective Photoreduction of CO_2_ into CO under Visible Light,” Green Chemistry, vol. 21, no. 24, pp. 6606, 2019.
